# Novel Antioxidant, Anti-α-Amylase, Anti-Inflammatory and Antinociceptive Water-Soluble Polysaccharides from the Aerial Part of *Nitraria retusa*

**DOI:** 10.3390/foods9010028

**Published:** 2019-12-26

**Authors:** Ilhem Rjeibi, Faiez Hentati, Anouar Feriani, Najla Hfaiedh, Cédric Delattre, Philippe Michaud, Guillaume Pierre

**Affiliations:** 1 Research unit of Macromolecular Biochemistry and Genetics, Faculty of Sciences of Gafsa, Gafsa 2112, Tunisia; rjeibii@yahoo.fr (I.R.); najla.hfaiedh@yahoo.fr (N.H.); 2 Unité de Biotechnologie des Algues, Biological Engineering Department, National School of Engineers of Sfax, University of Sfax, Sfax 3029, Tunisia; 3 Université Clermont Auvergne, CNRS, SIGMA Clermont, Institut Pascal, F-63000 Clermont-Ferrand, France; cedric.delattre@uca.fr (C.D.); guillaume.pierre@uca.fr (G.P.)

**Keywords:** *Nitraria retusa*, polysaccharides, physicochemical characterization, biological activities, mice

## Abstract

In this paper, water-soluble polysaccharides (named as NRLP) were extracted from *Nitraria retusa* leaves. The main structural features of NRLP were determined by High-pressure size exclusion chromatography, Fourier transform infrared and Gas Chromatography/Mass Spectrometry-Electronic Impact analysis. The in vitro and in vivo biological potential of NRLP were evaluated by measuring its antioxidant (•OH and DPPH• scavenging, total antioxidant capacity), anti-α-amylase as well as anti-inflammatory and antinociceptive activities in a mice model. NRLP was composed of Rha (33.7%), Gal (18.1%), GalA (15.0%), Glc (13.3%), Ara (13.3%), Xyl (3.8%), and GlcA (2.8%) and showed a Molecular Weight (Mw) of 23.0 kDa and a polydispersity index (PDI) of 1.66. The investigations highlighted a significant antioxidant activity (IC_50_ = 2.4–2.6 mg/mL) and an inhibition activity against α-amylase (IC_50_ = 4.55 mg/mL) in a dose-dependent manner. Further, NRLP revealed interesting anti-edematous effects and antinociceptive activities (both > 70%). These results open up new pharmacological prospects for the water-soluble polysaccharides extracted from *Nitraria retusa* leaves.

## 1. Introduction

Inflammation, a physiological feedback to tissue injury or surgical trauma, requires the participation of various inflammatory mediators, including histamine, nitric oxide, serotonin, interleukin, prostaglandins, and tumor necrosis factors, as well as a variety of cell populations (macrophages and neutrophils) [1]. Several papers have shown a relationship between oxidative damage, inflammation, and pain through the modulation of several immune cells and inflammatory mediators [2,3]. The authors reported that the agents or substances that counteract oxidative stress could help to alleviate inflammatory disorders. Non-steroidal anti-inflammatory drugs (NSAIDs) were commonly demonstrated to reduce inflammatory pains. Due to safety concerns associated with their use, research on new natural products, such as plant-derived polysaccharides, for the management of antinociceptive and anti-inflammatory disorders is of then utmost importance [4,5,6].

Polysaccharides have attracted attention due to their safety, biodegradability, and nontoxic effects [7,8]. Plant-derived polysaccharides are widely used because of their manifold biological properties. For instance, several studies have shown their anti-fatigue activities [9], anti-hypercholesterolemic effects [10], hepato-nephroprotective activities [4], anticoagulant effects [11] or antibacterial properties [12]. Recent data have also demonstrated that these natural biomolecules possess a considerable potential to prevent various oxidative stress-related disorders [13]. The therapeutic effects of natural antioxidant have been assigned to their abilities to inhibit the lipid peroxidation and to scavenge free radicals [14]. On the other hand, the inhibition of amylases, a key enzyme involved in carbohydrate digestion, has been reported to be an important target to decrease the hyperglycemia in diabetic patients. In this context, several works have been published on the efficiency of polysaccharides to inhibit amylases [15,16].

*Nitraria* spp., a salt-tolerant shrub plants, belongs to the Nitrariaceae family and is particularly distributed in Asia, China, Africa, Russia and Europe. Traditionally, plants of this genus are used for the treatment of diverse diseases. The leaves of *N. tangutorum* are used to prevent neuropathy and arrhythmia whereas its fruits are used to treat stomachache, dyspepsia, chronic fatigue syndrome and colds [17]. The fruits of *N. sibirica* are widely used to treat hypertension disorders [18]. Regarding the literature, *Nitraria* species contain numerous phytochemical constituents with multiple pharmacological activities such as hypoglycemic, hepatoprotective, antimutagenic, antimicrobial, hypotensive and antitumor capacities [19]. Among the *Nitraria* species, only *N. retusa* grows widely in the southern part of Tunisia and it is popularly known as Ghardaq. The use of *N. retusa* in folk medicine involves the treatments of inflammation [20]. Several reports evinced that the leaf of *N. retusa* contains high amounts of flavonoids, including different isomers of isorhamnetin, which are responsible for the strong scavenging activities in vitro, as well as, anti-proliferative and anti-genotoxic effects towards Caco-2 and K562 cell lines, respectively [21]. It has been also demonstrated that aqueous fruit extracts exhibit protective effects against penconazole-induced nephrotoxicity in rats [22]. Recently, Boubaker et al. [23] reported the strong immunomodulation properties and antitumoral activities of the chloroform extract of *N. retusa* leaves owing to palmitic acid and β-sitosterols. Our latest study revealed that polysaccharides from *N. retusa* fruits have in vitro antioxidant activity and significant in vivo hypolipidemic, hepatoprotective, and cardioprotective effects [24].

To our knowledge, there have been no studies dealing with the chemical elucidation and pharmacological potentials of water-soluble polysaccharides from its leaves. Some structural features of NRLP were determined by high pressure size exclusion chromatography (HPSEC), Fourier transform infrared (FTIR) spectroscopy and gas chromatography coupled to mass spectrometry (GC/MS) analysis. The antioxidant (•OH and DPPH• scavenging, total antioxidant capacity), α-amylase inhibitory effects, and the anti-inflammatory and antinociceptive activities in a mice model were also monitored on NRLP.

## 2. Materials and Methods 

### 2.1. Plant Material

*Nitraria retusa* leaves were harvested from Tabeddit, Gafsa (34°25′60″ N latitude and 8°16′0″ E longitude), in June 2017. Voucher specimen (MSE-0759) was authenticated at Botany department, Faculty of Sciences Gafsa, Tunisia and deposited at the herbarium. The leaves were washed, dried under shade, grounded to a fine powder (< 50 mesh) and stored at room temperature for further research.

### 2.2. Extraction and Purification of NRLP from N. retusa Leaves 

Polysaccharides from *N. retusa* leaves were extracted as described by Rjeibi et al. [24]. The powdered leaves (50 g) were extracted for 60 min at room temperature using 95% ethanol to eliminate lipids. The dried residue was extracted in hot water (450 mL) with magnetic stirring (450 rpm) for 2 h at 100 °C. The polysaccharide obtained after precipitation with cold (−20 °C) anhydrous ethanol (3/1, *v*/*v*) was solubilized in ultra-pure water and deproteinised by Sevag reagent (4 volumes of chloroform/1 volume of n-butanol). The solution was precipitated with 95% ethanol for 24 h at 4 °C, and then centrifuged at 2300× *g* for 10 min. The obtained fraction was dialyzed (cut-off 3500 Da) against ultra-pure water for three days at 4 °C with conductimetry monitoring. The precipitate was solubilized (30 g/L) in distilled water and freeze dried at −55 °C for 24 h (Alpha1-2 LD plus, Martin Christ, Germany) to obtain the enriched polysaccharides fraction (NRLP).

### 2.3. Preliminary Structural Features of NRLP

#### 2.3.1. Biochemical Composition

The total carbohydrate was determined by the phenol-sulfuric acid procedure of Dubois et al. [25], using Glc as standard. The protein content was determined using the Bradford method [26] and the bovine serum albumin as a standard. The total phenolic compounds, total neutral sugar and uronic acid contents were determined using respectively the Folin-Ciocalteu method [27], sulfuric resorcinol method [28] and m-hydroxydiphenyl test [29]. The conversion of conductivity into NaCl content was done assuming that 2 mS/cm were equivalent to 1 g/L of NaCl.

#### 2.3.2. FT-IR Spectroscopy Analysis

FT-IR spectra of NRLP were recorded on a VERTEX 70 FT-IR instrument equipped with an ATR A225 diamante (Bruker VERTEX 70, Ettlingen, Germany). Fifty scans were recorded at room temperature (reference against air) ranging from 4000 to 400 cm^−1^. The data were analyzed using OPUS 7.2 software (Bruker, Ettlingen, Germany).

#### 2.3.3. Determination of Average Molecular Weight

HPSEC was used to investigate the molecular weight and homogeneity of NRLP. The apparatus consisted of HPLC (HPLC 1100 series, Agilent, Palo Alto, CA, USA) equipped with a differential refractive index detector (RIDG1362, Agilent). Two columns (TSKgel PW_XL_ type Guard column 5000 and 3000, Tosoh Bioscience GMBH, Stuttgart, Germany) were eluted with Sodium nitrate (NaNO_3_, 0.1 M) at 40 °C with a volume flow of 0.8 mL/min, using pullulan standards for calibration (from 1.3 to 800 kDa, 10 g/L). A solution of NaNO_3_ (0.1 M) was used to solubilize NRFP (10 g/L) for 24 h under magnetic stirring before injection (20 µL). The number (Mn) and weight (Mw) average molecular weights as well as the polydispersity index (PDI) were calculated using Equations (1)–(3):

(1) $$M_{n}=\frac{\sum N_{i}M_{i}}{\sum N_{i}}$$

(2) $$M_{w}=\frac{\sum N_{i}{M_{i}}^{2}}{\sum N_{i}M_{i}}$$

(3) $$PDI=\frac{M_{w}}{M_{n}}$$

where *M_i_* and *N_i_* are, respectively, the molecular weight and number of moles of polymer species.

#### 2.3.4. Analysis of Monosaccharide Composition

The monosaccharide composition of NRLP was determined using GC/MS-EI according to Pierre et al. [30]. Briefly, 10 mg of NRLP were hydrolyzed with 2 M trifluoroacetic acid (TFA) at 120 °C for 90 min. After the derivatization of hydrolysates using BSTFA: TMCS (99:1), the mixture was evaporated under nitrogen steam at 60 °C and trimethylsilyl-*O*-glycosides were solubilized into dichloromethane. The samples were injected on an Agilent 6890 GC system coupled to an Agilent 5973 Network Mass Selective Detector (Agilent, Santa Clara, CA, USA), equipped with an OPTIMA-1MS column (Macherey-Nagel; 30 m, 0.32 mm, 0.25 μm). The apparatus was set up according to the following parameters, i.e., target ion: 40–800 m/z, injector line temperature: 250 °C, trap temperature: 150 °C, split ratio: 50:1, helium pressure: 8.8 psi; helium flow rate: 2.3 mL/min, ionization: 70 eV by electronic impact. The rise in temperature was a first step at 100 °C during 3 min, an increment of 8 °C/min up to 200 °C for 1 min and then a final increment of 5 °C/min up to 215 °C. The monosaccharide standards (Glc, Ara, GlcA, Fuc, Rha, Gal, Man, Xyl and GalA) were prepared using the same procedure. 

### 2.4. Biological Properties of NRLP

#### 2.4.1. In Vitro Antioxidant Activities 

##### Total Antioxidant Capacity 

This test was assayed according to Prieto et al. [31]. Accurately 0.1 mL of NRLP solution (0.5, 1, 1.5, 2, 2.5, and 3 mg/mL) was mixed with 0.6 mM sulfuric acid, 28 mM sodium phosphate and 4 mM ammonium molybdate at 95 °C for 90 min. After cooling, the final absorbance was measured at 695 nm. Ascorbic acid and butylated hydroxytoluene (BHT) were used as positive controls.

##### Hydroxyl Radical Scavenging Ability

Hydroxyl radical (•OH) scavenging activity was assayed using the method of Zhong et al. [32]. Briefly, 1 mL of different concentrations of NRLP (0.5, 1, 1.5, 2, 2.5, and 3 mg/mL) was incubated with 1 mL of FeSO_4_ (9 mM) and 1 mL of hydrogen peroxide (9 mM) in 1 mL of salicylic acid-ethanol (9 mM) at 37 °C for 30 min. The absorbance of samples was recorded at 510 nm, using ascorbic acid and BHT as positive controls. The hydroxyl radical scavenging activity was estimated using Equation (4).

(4) $$\mathrm{Inhibition}\;\left(\%\right)=\frac{\mathrm{Absorbance}\;\mathrm{of}\;\mathrm{control}-\mathrm{Absorbance}\;\mathrm{of}\;\mathrm{sample}}{\mathrm{Absorbance}\;\mathrm{of}\;\mathrm{control}}\times 100$$

##### DPPH Radical Scavenging Assay

The antioxidant activity of NRLP on DPPH radical was performed using the procedure of Bounatirou et al. [33] with slight modifications. Briefly, 500 µL of different concentrations (0.5, 1, 1.5, 2, 2.5, and 3 mg/mL) of NRLP were mixed with methanol (375 µL) and DPPH methanolic solution (125 µL, 0.02%) and incubated for 30 min in the dark at room temperature. The control tube was prepared using all reagents except the polysaccharide while ascorbic acid and BHT were used as the positive controls. The absorbance was recorded at 517 nm. NRLP scavenging activity on DPPH radical was estimated according to Equation (5).

(5) $$\mathrm{Inhibition}\;\left(\%\right)=\frac{\mathrm{Absorbance}\;\mathrm{of}\;\mathrm{control}-\mathrm{Absorbance}\;\mathrm{of}\;\mathrm{sample}}{\mathrm{Absorbance}\;\mathrm{of}\;\mathrm{control}}\times 100$$

#### 2.4.2. α-Amylase Inhibitory Assay 

The anti-α-amylase activity of NRLP was conducted as described by Kwon et al. [34] with minor modifications. Briefly, 500 µL of different concentrations (0.5, 1, 3, 6, 9, 12 mg/mL) of NRLP prepared in PBS were mixed with α-amylase (500 µL, 1.0 U/mL) and incubated for 10 min at 37 °C. Then, potato starch solutions (500 µL, 1%) was added to the mixture and re-incubated for 10 min at 37 °C. Finally, the reaction was stopped using 1 mL of dinitrosalicylic acid (DNS) reagent and heated for 5 min in a boiling water bath. The absorbance of the resulting mixture was measured at 520 nm after dilution with 10 mL of distilled water. The control tube was prepared using all reagents except the polysaccharide, while acarbose was used as the positive control. The inhibitory activity was estimated regarding Equation (6).

(6) $$\mathrm{Inhibition}\;\left(\%\right)=\frac{\mathrm{Absorbance}\;\mathrm{of}\;\mathrm{control}-\mathrm{Absorbance}\;\mathrm{of}\;\mathrm{sample}}{\mathrm{Absorbance}\;\mathrm{of}\;\mathrm{control}}\times 100$$

#### 2.4.3. In Vivo Pharmacological Properties 

##### Animals and Toxicity Test

Swiss albino mice of about 26–29 g were used. Animals were cared according to the Tunisian code of practice for the Care and Use of Animals for Scientific Purposes and the European convention for the protection of vertebrate animals used for experimental and other scientific purposes (Council of Europe No 123, Strasbourg, 1985). Approval for this study was received from the Ethics Committee of the Faculty of Sciences of Gafsa, Tunisia on January 9th, 2018 according to the Medical Ethical Committee for the Care and Use of Laboratory Animals of Pasteur Institute of Tunis (Approval No: LNFP/Pro 152012).

The oral toxicity of NRLP was tested using four oral doses (100, 200, 400, 800 mg/kg, p.o., *n* = 6), while the control group was treated with sterile saline (0.9% NaCl, 2.5 mL/kg, p.o.). Treated animals were observed for toxic symptoms and death rate for 12 h, 24 h and 14 days.

In the following pharmacological experiments, mice (*n* = 6 per group) were treated 60 min before each test with NRLP (100 and 400 mg/kg BW, p.o.), sterile saline solution (0.9% NaCl, 2.5 mL/kg, p.o.) and two NSAIDs, i.e., indomethacin (10 mg/kg, p.o.) and paracetamol (100 mg/kg, p.o.).

##### Antinociceptive Activity

###### Hot Plate Test

Treated animals were placed into a glass beaker on the heated plate at 50 ± 0.5 °C for a maximum of 40 s to prevent paw damages. The reaction time was noted when the mice licked their paws and that during 0, 30, 60, 90, and 120 min post-treatment [35]. The inhibitory activity was estimated according Equation (7).

(7) $$\mathrm{Inhibition}\;\left(\%\right)=\frac{T_{n}-T_{0}}{T_{0}}\times 100$$

where T_n_ is the reaction time following the administration of NRLP or the indomethacin and T_0_ was the initial reaction time.

###### Writhing Test

Acetic acid solution (1%, *v*/*v* in saline, 10 mL/kg) was injected intraperitoneally and the number of writhes from 10–30 min of post-injection was enumerated [36]. The inhibitory activity was estimated using Equation (8).

(8) $$\mathrm{Inhibition}\;\left(\%\right)=\frac{W_{c}-W_{t}}{W_{c}}\times 100$$

where W_t_ is the average number of writhes of the groups treated with NRLP or paracetamol and W_c_ is the average number of writhes of the control animals.

###### Formalin Test

Formalin (1% *v*/*v*; 20 µL) was administered subcutaneously in the hind paws of mice. The time, in second, in which animals licked their paws in response to chemical stimuli, was recorded during the first 5 min (neurogenic phase) and from 15 to 30 min (inflammatory phase) [37]. The inhibitory activity was calculated regarding Equation (9).

(9) $$\mathrm{Inhibition}\;\left(\%\right)=\frac{T_{c}-T_{t}}{T_{c}}\times 100$$

where T_t_ is the reaction time of the groups treated with NRLP or the indomethacin and T_c_ is the reaction time of the control group. 

##### Anti-Inflammatory Activity

###### Carrageenan-Induced Mice Paw Edema

Carrageenan (1% carrageenan in 0.9% NaCl, 50 µL/paw), was administered in the plantar right-hind paws of the mice to induce inflammation. The progression of paw edema was followed by the measurement of the paw diameter of each mouse at 0, 1, 2, 3, 4 and 5 h of inflammatory induction, using a dial caliper [38].

After the last measurement of paw volume, animals of each group were killed by cervical decapitation to shun stress. The liver tissues and the right hind paw were quickly removed, and then were washed in ice-cold 1.15% potassium chloride solution, homogenized into 2 mL ice-cold buffer (pH 7.4) and centrifuged for 30 min at 5000 rpm and 4 °C. The supernatants were collected and stored at −20 °C until their uses.

###### Carrageenan-Induced Mice Lipid Peroxidation Levels

To estimate the degree of lipid peroxidation in the liver and paw tissues, 100 µL of homogenate supernatants were added to 100 µL of trichloroacetic acid (TCA, 5%) and the mixture were centrifuged at 4000× *g* for 10 min. After that, 100 µL of the supernatant and 200 µL of thiobarbituric acid reagent (TBA, 0.67%) were incubated for 15 min on a boiling water bath. The level of lipids peroxidation was measured as thiobarbituric acid reactive substances (TBARS) and was calculated as malondialdehyde (MDA) formation, according to Draper and Hadley [39].

### 2.5. Statistical Analysis

Statistical analysis was performed by PASW Statistics Version 18.0 (SPSS Inc, Chicago, IL, USA) using one-way analysis of variance (ANOVA) followed by the Tukey’s test. The differences were considered significant at *p* < 0.05.

## 3. Results and Discussion 

### 3.1. Chemical Characterization of NRLP

**Figure 1:**
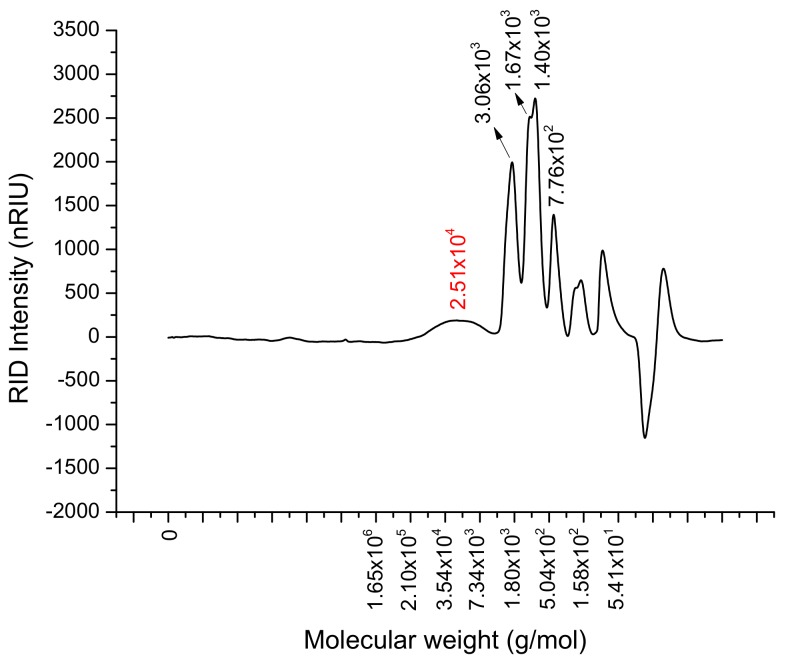
High-pressure size exclusion chromatography (HPSEC) chromatogram of polysaccharides from *N. retusa* leaves (NRLP, 10 g/L) in 0.1 M NaNO_3_.

NRLP was obtained from *N. retusa* leaves. The extraction yield of NRLP was about 4% (*w*/*w*), which was lower than those obtained from their fruits (8.7%, *w*/*w*) and olive leaves (7.2% *w*/*w*) [40,41,42,43] but better than that for *Malva aegyptiaca* leaves (1.46%) [12]. The biochemical analysis showed that NRLP was composed of approximately 18% of uronic acid, 59.7% of total sugars and 17.3% of protein (Table 1). High protein content was previously reported for polysaccharides extracted from *M. aegyptiaca* leaves (17.14%), *N. retusa* fruits (18.67%) and *Citrus aurantium* (21%) [12,24,41]. The authors suggested that high protein levels could be related to the linkage between protein and polysaccharides by hydrophobic interaction and hydrogen and covalent bonds. The molecular weight of NRLP was 23.0 × 10^3^ g/mol (Figure 1) with a PDI value of 1.66 (Table 1). It was reported that the small molecular weight of polysaccharides could help to enhance their biological activities [42,43].

**Figure 2:**
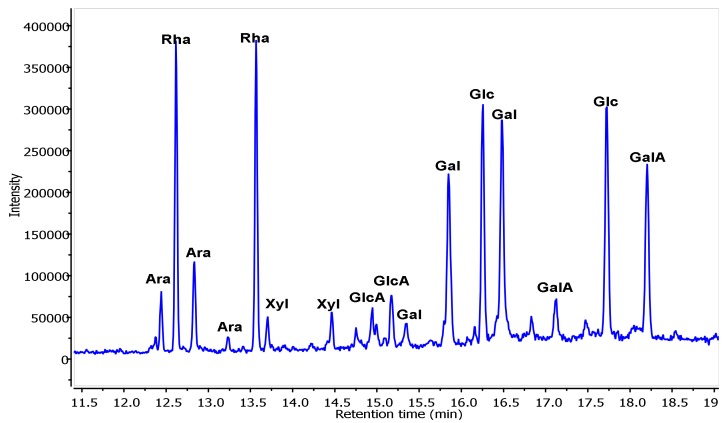
Gas Chromatography/Mass Spectrometry - Electronic Impact (GC/MS-EI) (70 eV) total ion chromatogram of trimethylsilyl-*O*-glycoside residues prepared from NRLP.

The monosaccharide composition (Figure 2, Table 1) indicated that NRLP is constituted of Rha (33.7%), Gal (18.1%), GalA (15.0%), Glc (13.3%), Ara (13.3%), Xyl (3.8%) and GlcA (2.8%). NRLP was a heteropolysaccharide, probably mainly composed of a pectin-like structure which was similar with previous studies [44]. Glc and Xyl could be some contaminants from cell-wall polysaccharides such as cellulose and hemicellulose [45]. The PDI value (1.66) seemed in accordance with this statement about cell-wall contamination due to the presence of very low oligosaccharides (around 10^3^ Da). The monosaccharide composition of NRLP advised that rhamnogalacturonan I (RG I), homogalacturonan (HG), and arabinogalactan (AG II) might exist in NRLP from *Nitraria* leaves [44,46,47]. Although the NRLP monosaccharide composition was similar to those of previously reported pectic polysaccharides, there are some differences. For example, a pectic polysaccharide from *Castanea henryi* was composed of Man (10.70%), Rha (8.70%), GalA (38.21%), Gal (13.75%) and Ara (28.63%) [48]. Jayaram et al. [49] reported the presence of Gal (54%), Ara (20%), Rha (1%), Xyl (3%), Man (4%) and Glu (18%) in the monosaccharide composition of the pectic polysaccharide from *Zea mays*.

### 3.2. ATR-FTIR Spectroscopy

**Figure 3:**
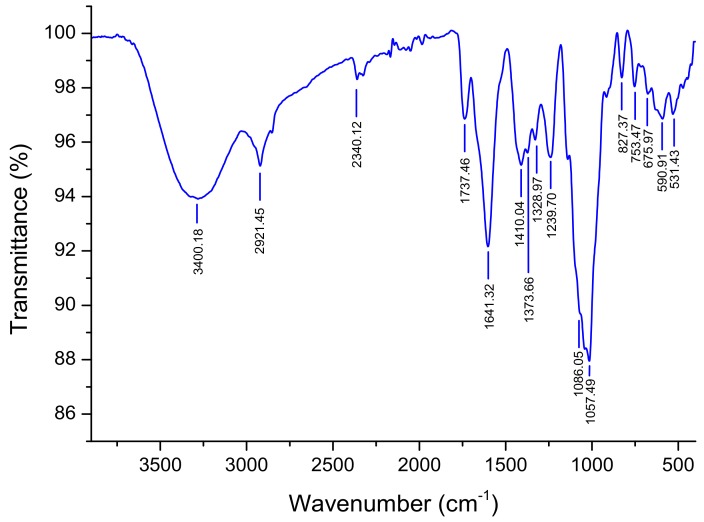
FTIR spectrum of NRLP.

The infrared spectrum of NRLP is shown in Figure 3. The intense absorption band at 3400 cm^−1^ corresponded to -OH stretching vibrations from polysaccharide and water. The signals around 2921, 1641, 1230 and 1057 cm^−1^ were assigned to carbonyl ester (C=O) groups and -CH stretching band of polysaccharides composing d-Xyl, d-Glc and d-GalA [50] and the peak at 1086 cm^−1^ showed the presence of d-Gal [46]. The characteristic bands at 1641 and 1410 cm^−1^ belonging to the stretching vibration of -COOH of uronic acid [51]. The two bands at 1086 and 1057 cm^−1^ suggested a pyranose form for the carbohydrates constituting NRLP [52]. The absorption band at 827 cm^−1^ indicated the possible linkage of *α*-glycosides [53].

### 3.3. Antioxidant Activities of NRLP

Regarding the literature, the polysaccharides isolated from natural sources, have been demonstrated to own potent in vitro antioxidant ability [24,39]. In their comprehensive review, Wang et al. [54] explained that the underlying mechanisms for in vitro antioxidant results are hard to address. For example, the chelation activity of polysaccharides is involved in antioxidant properties and can be attributed to specific groups such as -OH, -O-, -SH, -PO_3_H_2_, -C=O, -COOH, -NR_2_, or -S- [55]. Specific side chains, such as 1→2, 1→4 or 1→6, can also change these activities as well as Rha, Fuc or Man residues. Besides, cationic and anionic functional groups, such as uronic acids, are also considered as factors that may affect the antioxidant activity of polysaccharides [56]. Low molecular weight polysaccharides and/or oligosaccharides possess the best antioxidant activities due to the higher ratio of reducing terminal units [42].

**Figure 4:**
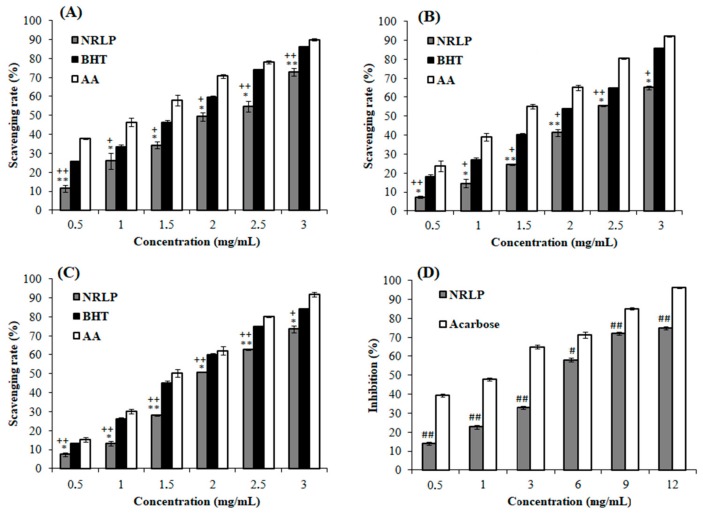
Antioxidant capacity of NRLP evaluated using (**A**) phospho-molybdenum, (**B**) hydroxyl and (**C**) DPPH radicals scavenging assays in different solutions and (**D**) the α-amylase inhibition effect. Data are shown as means of three separate experiments. * *p* < 0.1 and ** *p* < 0.01 compared with the positive control ascorbic acid (AA). ^+^ *p* < 0.1 and ^++^ *p* < 0.01 compared with the positive control butylhydroxytoluene (BHT). ^#^ *p* < 0.05 and ^##^ *p* < 0.01 compared with the positive control acarbose.

The total antioxidant capacity of NRLP measured using phosphomolybdenum assay is shown in Figure 4A. An increase of the inhibitory effect of NRLP (11.5–72.9%) was detected at all the concentration range (0.5–3 mg/mL). However, the reducing activities of BHT (25.6–86.0%) and ascorbic acid (37.8–90.0%) were higher for the same concentration range. Such results have been reported for other polysaccharides isolated from *Mentha piperita* leaves [57]. The decent antioxidant activity of NRLP (IC_50_ = 2.03 mg/mL) was 3.85-fold more efficient than the one reported for polysaccharides extracted from *N. retusa* fruits (IC_50_ = 7.83 mg/mL) [24].

•OH are classified as the means noxious reactive oxygen species which induces tissue injuries. As shown in Figure 4B, the elimination of •OH by NRLP was concentration dependent. At 3 mg/mL, the inhibition abilities of NRLP, BHT, and ascorbic acid were 65.2%, 85.8% and 92.2%, respectively. NRLP showed moderate •OH scavenging ability (IC_50_ = 2.42 mg/mL), compared to some polysaccharides extracted from *N. tangutorum* (IC_50_ = 0.82 mg/mL) [58]. Similar observations were previously reported for polysaccharide extracted from *Lupinus luteus* (IC_50_ = 2.50 mg/mL) [59]. In general, •OH scavenging activities of polysaccharides were affiliated to the presence of OH in their structures [60].

DPPH• is a highly stable and useful lipophilic free radical for evaluating free radical scavenging abilities of antioxidant from natural chemical compounds like polysaccharides [4,5,16]. The scavenging activities of these antioxidants were linked to their hydrogen-donating abilities. As illustrated in Figure 4C, the maximum scavenging activities of NRLP, ascorbic acid, and BHT were 73.5%, 91.9%, and 84.2%, with IC_50_ values of 2.7 mg/mL, 1.5 mg/mL, and 1.7 mg/mL, respectively.

It is important to remember that there is a high correlation between the antioxidant ability of polysaccharides and their structures. The determining agents of this correlation could be the degree of branching, linkage, molecular weight, monosaccharide composition, and conformation [56,61]. Rha and GalA residues composing NRLP should be also responsible to these activities. The scavenging ability is directly related to the uronic acid content of polysaccharides [54]. Luo et al. [60] also reported that the polysaccharide DNP4-2 extracted from *Dendrobium nobile* with the higher rhamnose content (12.6%) showed the strongest scavenging effect on free radicals, compared to DNP3-1 (3.76% Rha) and DNP1-1 (2.11% Rha). Overall, all the antioxidant values of NRLP are too low to compete with the most valuable antioxidants. For newcomers, an IC_50_ under 100 µg/mL should be the upper limit for considering significant •DPPH or •OH antioxidant activities, for example.

### 3.4. NRLP with α-Amylase Inhibitory Effect

α-amylase play an important role in carbohydrate hydrolysis and incorporation. Thus, the inhibition of this enzyme will delay the degradation of the composite sugars such as starch and extend overall carbohydrate digestion time. Additionally, controlling glucose production from food sources following α-amylase inhibitor consumption could be a good strategy for management of hyperglycemia as well as type 2 diabetes mellitus [16,62]. The inhibitory effect of NRLP on α-amylase is illustrated in Figure 4D.

The results showed that NRLP exhibited concentration-dependent effects on α-amylase inhibition from 0.5 to 12 mg/mL. At 12 mg/mL, NRLP showed the maximum inhibitory activity with an IC_50_ value of 4.55 mg/mL, which was about 4 times lower than the positive control acarbose (IC_50_ = 1.04 mg/mL). Similarly, Yan et al. [15] stated that the polysaccharide from *Corbicula fluminea* exhibited significant α-amylase inhibition activity with an IC_50_ close to 4.9 mg/mL. The inhibitory effect of NRLP was considerably better than those reported for other plants used as hypoglycemic agents for diabetics, like the polysaccharides from *Cucurbita moschata* which showed 41.3% of inhibition of α-amylase at a concentration of 5 mg/mL [16]. It should be kept in mind that beyond the IC_50_ value, the molar mass (Mw) of the tested polysaccharide is particularly important. Thus, oligosaccharides from this polymer should possess a better α-amylase inhibition activity at lowest degree of polymerization. Further experiments could be proposed to determine the real potential of NRLP and its low molecular weight forms as a hypoglycemic agent.

### 3.5. Antinociceptive Activity 

**Figure 5:**
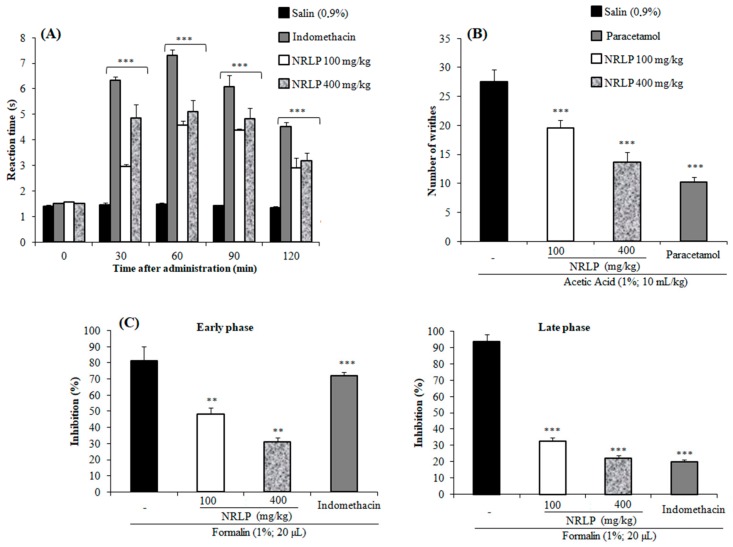
Antinociceptive activity of NRLP (100 and 400 mg/kg) and indomethacin (10 mg/kg) evaluated using (**A**) hot-plate, (**B**) acetic acid tests. (**C**) The formalin test was measured during the early phase (0–5 min) and late phase (15–30 min). All data are expressed as mean ± SD (*n* = 6). *** *p* < 0.001 and ** *p* < 0.01 when compared with the normal control groups.

Firstly, the antinociceptive activity of NRLP was explored using the hot plate assay. This model evaluates the effects of thermal stimulation to skin tissues and analyses the possible central action by measuring the jumping or licking time [63]. As shown in Figure 5A. pre-treatment of the mice with NRLP (100 and 400 mg/kg) revealed considerable anti-nociceptive effects by prolonging the latency time. At 60 min of post-treatment, NRLP reached a maximum increase in the latency to the thermal stimulus with 4.57 s (at 100 mg/kg) and 5.11 s (at 400 mg/kg) compared to the control (1.48 s). Similarly, the maximum of latency to the thermal stimulus in mice treated with the standard drug, indomethacin (10 mg/kg), was observed at 60 min. These findings evinced that NRLP might exert its anti-analgesic potency through the central nervous mechanisms. A similar effect has been highlighted for the polysaccharides extracted from *Suaeda fruticosa* [5] and *Morinda citrifolia* for example [6].

To evaluate the possible peripheral analgesic effect of NRLP, a chemical stimulus (acetic acid) was employed. It has long been demonstrated that acetic acid administration decreased the writhing response and increased writhing numbers through the activation of mitogen-activated protein kinase and the release of chemical mediators (tumor necrosis factor-α, serotonin, and prostaglandins) [64,65]. As shown in Figure 5B, the pre-treatment of the mice with NRLP at the doses of 100 and 400 mg/kg, significantly reduced the number of abdominal constrictions with inhibition values of 28.6% and 50.6%, respectively. Similarly, pre-treatment of mice with paracetamol (100 mg/kg, p.o.) showed significant antinociceptive effects with an inhibition rate of 62.8%, compared to the control group. These data corroborate the results obtained for other polysaccharides in which the analgesic action has been affiliated to the blockage of the release of various mediators in response to chemical stimulus [5,66].

To further confirm the antinociceptive effects of NRLP, the formalin test was performed. This model evaluates the possible effects of natural products into neurogenic (early phase) and inflammatory pains (late phase). The early phase is a result of direct nociceptors activation (the sensory C fibers), and the late phase is a consequence of the production of inflammatory mediators, which stimulates peripheral nociceptors [37]. As shown in Figure 5C, pre-treatment of mice with indomethacin (10 mg/kg, p.o.) reduced the licking time only in the late phase of the formalin test with inhibition rate of 78.90%. Note that the antinociceptive effects of NRLP were observed in the two phases with predominant action in the second phase. At 400 mg/kg, NRLP significantly inhibited the neurogenic and inflammatory phases of formalin-induced pain with inhibition of 60.0% and 76.4%, respectively, which confirms the central and peripheral analgesic effects of this bioactive polysaccharide. Antinociceptive effects of polysaccharides can be assigned to their OH radical scavenging abilities since the accumulation of this oxygen free radical will increase the content of calcium into cells, which plays a fundamental role during the induction of nociceptive response [5,66,67].

### 3.6. Anti-Inflammatory Activity

**Figure 6:**
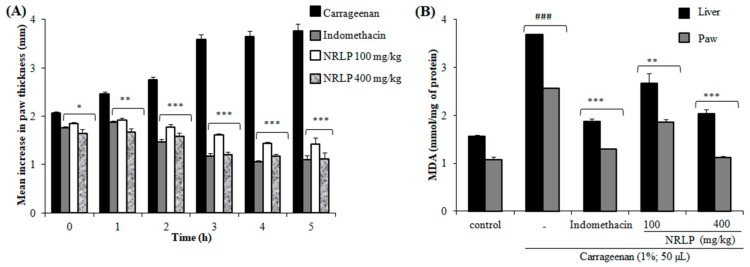
Effect of NRLP (100 and 400 mg/kg) and indomethacin (10 mg/kg) on carrageenan-induced mice paw edema (**A**) and MDA concentrations in paw edema and liver (**B**). Each column represents mean ± SD of six mice per group. ^###^ *p* < 0.001 compared with the control group. * *p* < 0.05, ** *p* < 0.01 and *** *p* < 0.001 compared with the carrageenan group.

The carrageenan-induced paw edema test was carried out to determine the anti-inflammatory effects of NRLP. It is known that intraplantar injection of carrageenan involves the liberation of serotonin and histamine in the first phase (1 h after carrageenan administration), release of kinins in the second phase (≈after 2 h) and finally the induction of cyclooxygenase (COX-2) as well as the release of prostaglandins (PGE2) in the third phase (≈after 3 h) which results in paw edema formation [68]. In this study, the pre-treatment of mice with NRLP at 100 mg/kg and 400 mg/kg significantly reduced the three stages of inflammation with a maximum inhibition of 68.0% and 71.9%, respectively at the 4th h after carrageenan injection (Figure 6A). Pre-treatment of mice with indomethacin (10 mg/kg, p.o.) decreased the edematous response with a maximum inhibition percentage at the 5th h (73.9%). Such findings suggest that the anti-inflammatory effects of NRLP are associated with the inhibition of histamine and prostaglandin synthesis, as well as, the inhibition of the elevated production of the COX-2. There is also research that describes the role of polysaccharides in the management of these disorders. It has also been reported that the polysaccharides from *Apium graveolens* have potential anti-inflammatory effects associated with the inhibition of the high production of pro-inflammatory cytokines [69]. The polysaccharides extracted from *Caesalpinia ferrea* and *Azadirachta indica* have also been demonstrated to inhibit the edematogenic effect of serotonin, prostaglandin (PGE2), and nitric oxide [6,70,71]. De Oliveira et al. [72] have demonstrated that the anti-inflammatory effects of polysaccharides from *Sedum dendroideum* leaves were attributed to reducing TNF-α and IL1-β secretion.

The inflammation process is also associated with the generation of free radicals which contribute to the edema formation [73]. Excessive production of ROS leads to oxidative damage and promotes lipid peroxidation of the membrane. In experimental studies, lipid peroxidation is assayed by measuring the MDA levels. The concentrations of MDA in both paw and liver tissues in mice treated by the carrageenan are illustrated in Figure 6B. This level was significantly elevated in the inflammatory group compared to the control group. However, the pre-treatment with NRLP at 100 mg/kg and 400 mg/kg showed a significant reduction in MDA levels in both tissues compared to the carrageenan group. Altogether, these findings suggested that NRLP may be selected as an alternative pharmacological drug for the management of inflammatory disorders.

## 4. Conclusions

The physicochemical characterization, antioxidant, hypoglycemic and anti-inflammatory properties of a novel polysaccharide NRLP from *N. retusa* leaves were investigated. Results revealed that NRLP was composed of Rha (33.7%), Gal (18.1%), GalA (15.0%), Glc (13.3%), Ara (13.3%), Xyl (3.8%) and GlcA (2.8%) and had an average molecular weight of 23.0 × 10^3^ g/mol with a PDI value of 1.66. NRLP present decent total antioxidant activity (IC_50_ = 2.03 mg/mL) and potent •OH (IC_50_ = 246.85 μg/mL) and DPPH (IC_50_ = 2.7 mg/mL) scavenging abilities. Moreover, NRLP had significant α-amylase inhibition effect (IC_50_ = 4.55 mg/mL) in vitro. NRLP significantly alleviated pain in mice (thermal and chemical stimuli), inhibited carrageenan-induced paw edema and decreased the production of MDA in liver and paw. The outcomes obtained afford a new bioactive base for the development of *N. retusa* polysaccharides as accessible phytochemicals against inflammation and pain.

The physicochemical characterization, antioxidant, hypoglycemic and anti-inflammatory properties of a novel polysaccharide NRLP from *N. retusa* leaves were investigated. Results revealed that NRLP was composed of Rha (33.7%), Gal (18.1%), GalA (15.0%), Glc (13.3%), Ara (13.3%), Xyl (3.8%) and GlcA (2.8%) and had an average molecular weight of 23.0 × 10^3^ g/mol with a PDI value of 1.66. NRLP present decent total antioxidant activity (IC_50_ = 2.03 mg/mL) and potent •OH (IC_50_ = 246.85 μg/mL) and DPPH (IC_50_ = 2.7 mg/mL) scavenging abilities. Moreover, NRLP had significant α-amylase inhibition effect (IC_50_ = 4.55 mg/mL) in vitro. NRLP significantly alleviated pain in mice (thermal and chemical stimuli), inhibited carrageenan-induced paw edema and decreased the production of MDA in liver and paw. The outcomes obtained afford a new bioactive base for the development of *N. retusa* polysaccharides as accessible phytochemicals against inflammation and pain.

## Figures and Tables

**Table 1:** Preliminary characterization of NRLP extracted from *Nitraria retusa* leaves.

Composition	Value
Total sugars (%, *w*/*w*)	59.7 ± 0.12
Neutral sugars (%, *w*/*w*)	41.68 ± 0.05
Uronic acids (%, *w*/*w*)	17.97 ± 0.12
Proteins (%, *w*/*w*)	17.28 ± 0.17
Phenolic compounds (%, *w*/*w*)	1.41 ± 0.01
Conductivity (μs/cm)	77.1
[NaCl] equation (%)	4.02
M_w_^a^ (g/mol)	23,060
M_n_^b^ (g/mol)	13,903
PDI ^c^	1.66
Monosaccharides ^d^ (mol%)	
Rhamnose (Rha)	33.66
Galactose (Gal)	18.05
Galacturonic acid (GalA)	15.03
Glucose (Glc)	13.34
Arabinose (Ara)	13.30
Xylose (Xyl)	3.79
Glucuronic acid (GlcA)	2.83

Values are means ± SD of three separate experiments. ^a^ M_w_: Weight average molecular weight was measured by HPSEC-DRI. ^b^ M_n_: Number average molecular weight was measured by HPSEC-DRI. ^c^ PDI: Polydispersity index M_w_/M_n_. ^d^ Monosaccharide composition was determined by GC/MS-EI.

## Abbreviations

HPSEC	High-pressure size exclusion chromatography;
GC/MS-EI	Gas Chromatography/Mass Spectrometry- Electronic Impact;
DPPH	2,2-diphenyl-1-picrylhydrazyl;
FT-IR	Fourier transform infrared;
BSTFA:TMCS	*N*,*O*-bis(trimethylsilyl)trifluoroa-cetamide:trimethylchlorosilane
